# Intense solar activity reduces urinary 6-sulfatoxymelatonin in patients with COPD

**DOI:** 10.1186/s12931-023-02390-w

**Published:** 2023-03-24

**Authors:** Carolina L. Zilli Vieira, Petros Koutrakis, Man Liu, Daniel J. Gottlieb, Eric Garshick

**Affiliations:** 1 Department of Environmental Health, Harvard T.H. Chan School of Public Health, 401 Park Drive, Landmark Center Room 420, Boston, MA 02115 USA; 2 Pulmonary, Allergy, Sleep and Critical Care Medicine Section, Veterans Affairs Boston Healthcare System, Boston, MA USA; 3 Harvard Medical School, Boston, MA USA; 4 Channing Division of Network Medicine, Brigham and Women’s Hospital, Boston, MA USA

**Keywords:** Intense solar activity, Urinary 6-sulfatoxymelatonin levels, Pulmonary disease, Circadian rhythm disruption, And diabetes mellitus

## Abstract

**Background:**

Little is known about the link between solar activity and variations in melatonin. In this study, we investigated if melatonin's major urinary metabolite, urinary 6-sulfatoxymelatonin (aMT6s), is lowest under periods of intense solar activity.

**Methods:**

We investigated associations between high-energy solar particle events [Coronal Mass Ejection (CME) mass, speed and energy] on creatinine-adjusted aMT6s (aMT6sr) concentrations in 140 patients with chronic obstructive pulmonary disease (COPD) using up to four seasonal urine samples (n = 440). Mixed effect models with a random intercept for each subject were used to estimate associations, including effect modification attributable to diabetes, obesity, and reduced pulmonary function.

**Results:**

Higher values of CME were associated with reduced aMT6sr concentrations, with stronger associations in patients with diabetes. An interquartile range (IQR) increase in natural log CME_speed_ averaged through two days before urine collection was associated with a reduction of 9.3% aMT6sr (95%CI: − 17.1%, − 0.8%) in aMT6sr. There was a greater reduction in aMT6sr in patients with diabetes (− 24.5%; 95%CI: − 35.9%, − 11.6%). In patients without diabetes there was no meaningful association (− 2.2%; 95%CI: − 12%, 8.4%). There were similar associations with CME_energy_ and CME_mass_. There was no effect modification attributable to reduced pulmonary function or obesity.

**Conclusions:**

This is the first study in patients with COPD to demonstrate strong detrimental impact of high-energy solar particle events on aMT6sr, with greater associations in patients with diabetes. Since melatonin is an anti-oxidant, it is possible that adverse effects of intense solar activity may be attributable to a reduction in circulating melatonin and that patients with both COPD and diabetes may be more susceptible.

**Supplementary Information:**

The online version contains supplementary material available at 10.1186/s12931-023-02390-w.

## Background

Melatonin (*N*-acetyl-5-methoxytryptamine) is a potent nocturnal antioxidant hormone and efficient immuno-inflammatory regulator, which is primarily synthetized in the pineal gland [1]. Melatonin is also produced by other organs such as the retina and the gastrointestinal tract [1]. It is well known that pineal gland function and melatonin secretion are modulated by the environmental light/dark cycle via the suprachiasmatic nucleus (SCN) in the hypothalamus [2]. SCN is the “master clock” that regulates the 24 h-circadian rhythm, including melatonin/cortisol rhythms, gene expression, and autonomic nervous system (ANS) function [2–8]. The disruption of 24-h circadian rhythm with concomitant reduction of melatonin synthesis and serum levels seem also to be modulated by solar and geomagnetic activity (SGA)-related visible and non-visible electromagnetic radiation [3]. This may be relevant clinically, as we have previously described reductions in pulmonary function attributable to SGA [9]. We have also observed additional adverse effects of SGA that include increases in blood pressure, atrial and ventricular arrhythmias, and increases in circulating biomarkers of systemic inflammation [10–13]. In this study, we hypothesize that intense solar activity reduce levels of urinary 6-sulfatoxymelatonin (aMT6s), thereby providing a biologic pathway with the potential to contribute to the adverse effects of SGA.

Circulating melatonin has a short half-life (~ up to 45 min) and is rapidly metabolized in the liver with the major enzymatic metabolite of melatonin (aMT6s), which highly correlates with plasma melatonin levels [13, 14]. Studies in animals have suggested a link between seasonal rhythmicity of aMT6s levels and ~ 11-year solar cycles [8]. Melatonin levels can also be affected by genetic factors, sex, pineal gland size, and modifiable factors such as seasonal changes, latitudinal locations, medications (e.g., β-blockers and non-steroidal anti-inflammatory drugs), sleep quality, depression, smoking status, and comorbidities including diabetes and cancer [2, 13–20]. Levels of melatonin vary throughout life, reaching highest levels in children younger than 4 years, and declining within age [2, 19]. In the lung, melatonin may mitigate respiratory disease severity by modulating pro-inflammatory cytokines such as interleukin1β and 6, and TNF-α, reducing oxidative stress [1, 13, 16]. A reduction in melatonin also can increase the risk of the development of type 2 diabetes mellitus by impairing insulin sensitivity and glucose tolerance [6, 7].

Understanding the impact of solar activity on melatonin levels is critical to understanding natural exposures that may affect health outcomes, particularly in association with oscillations of 11-solar cycles, even though the relationship between variation in solar activity and melatonin levels in humans has not been well established. To investigate this, we took advantage of previously collected urine samples in a cohort of patients with COPD [21, 22] to assess the link between short-term variation in solar activity intensity and melatonin excretion, using aMT6s concentrations to estimate melatonin levels. We also assessed effect modification of comorbidities (diabetes mellitus, obesity, and reduced pre-bronchodilator pulmonary function) on aMT6s.

## Methods

### Study subjects

Between 2013 and 2017, 143 patients with chronic obstructive pulmonary disease (COPD) were recruited at Veterans Affairs (VA) Boston Healthcare System from Eastern Massachusetts and vicinity to investigate the impact of particulate air pollution exposures [20–22]. Participants had up to 4 seasonal visits scheduled at least 2 weeks after therapy for a COPD exacerbation. Participants were former smokers with 10 pack-years or more of lifetime smoking, and had a ratio of post-bronchodilator forced expiratory volume in one second to forced vital capacity (FEV_1_/FVC) of < 0.70 at a screening visit or emphysema on chest computed tomography. Individuals with malignancies other than local skin or stable prostate cancer, a systemic inflammatory disease such as rheumatoid arthritis; or with unstable heart disease, were excluded. By study design, in order to substantially reduce exposure to sources of indoor combustion, we excluded patients who were current smokers or lived with a current smoker, or who had a major source of indoor air pollution (e.g., wood stove or fireplace, frequent burning of incense or candles). At each study visit spirometry pre- and post-bronchodilator was conducted [23], medication use was reviewed, and height and weight were measured. At study entry, participants were asked if they had ever being told by doctor that they had sleep apnea or diabetes. The study protocol was approved by Institutional Review Boards at VA Boston and Harvard Medical School. We obtained informed consent from all participants prior to study procedures.

### Urinary 6-sulfatoxymelatonin (aMT6s) assessment

Study visits occurred during daylight hours where the time of urine sample collection was noted [median 11:22 AM (interquartile range (IQR): 2 h 34 min), mean 11:35 AM (standard deviation: 1 h 51 min);]. Samples were put on ice and transported to the VA Boston core laboratory and frozen at − 80 °C. For this analysis aMT6s (ng/ml) was measured in these stored samples in duplicate by an ELISA assay (Alpco, Salem, NH) in the Department of Laboratory Medicine, Boston Children’s Hospital, Boston, MA, USA. The day-to-day variabilities of the assay at concentrations of 6.8, 95 and 248 ng/mL are 11.0, 6.3 and 5.2%, respectively. The assay is sensitive to 1.0 ng/mL. To account for differences in urinary dilution, we measured urinary creatinine (mg/ml), using the ratio of aMT6s and creatinine (aMT6sr) as the study outcome (in ng/mg creatinine).

### Solar activity parameters

Parameters of solar activity events [corona mass ejection (CME)] were obtained from the NASA SOHO/LASCO CME (https://cdaw.gsfc.nasa.gov/CME_list/). CME is high-energy plasma ejected from the outer surface of the Sun that interacts with the earth’s magnetic field, producing geomagnetic disturbances and increased electromagnetic phenomena in the earth’s systems (e.g. atmosphere, geosphere). CME can take hours to days to reach earth depending on its energy, mass and speed. We converted hourly CME data in UTC time to local Boston time, and created daily averages. Daily CME data included the mean of CME_mass_ (grams of solar mass), CME_energy_ [in *erg*, *unit* of energy equal to 10^−7^ J (100 nJ)], and CME_speed_ (km/s). CME can occur many times per day or none, depending on the intensity of solar activity (higher intensity of solar activity results in an increased number of CME events). Because the CME values can range from negligible to trillions of units of CME_mass_, energy and speed, we used the natural log of CME variables.

### Statistical analysis

We used mixed effect models with random intercept for each subject to analyze the association of solar activity on the natural log transformed aMT6sr to normalize its distribution and meet model assumptions. As CME can take hours to days to reach Earth, we analyzed five windows of moving averages of exposure from the day of urine collection (day 0) to up to 4-days (day 4) *prior* to the urine sample collection.

The primary models included a single exposure variable (corona mass ejection energy, speed or mass) and covariates. Melatonin can be affected by sex, seasonal changes, medications (including β-blockers and non-steroidal anti-inflammatory drugs), sleep apnea, and comorbidities including diabetes [2, 13–20]. Therefore, model covariates included a priori were race (white vs. other), sex (male/female), age, body mass index (BMI), beta blocker use and non-steroidal anti-inflammatory medication use within 1 day of urine collection, diabetes, time of urine collection, history of sleep apnea, and season (winter, spring, summer, fall), and BMI.

$${aMT6sratio}_{ID} \sim {X}_{1 lCME\left[X\right]}+{X}_{2}{\mathrm{urine}}_{\mathrm{season}}+{X}_{3}\mathrm{sex}+{X}_{4}\mathrm{bmi}+{X}_{5}\mathrm{age}+{X}_{6}\mathrm{race} +{X}_{6}\mathrm{med}1 \left(\mathrm{NSAID}\right)+{X}_{7}\mathrm{med}2 \left(\mathrm{beta blockers}\right)+{X}_{8}{\mathrm{baseline}}_{\mathrm{diabetes}}+{X}_{9}{\mathrm{baseline}}_{\mathrm{apnea}}+{X}_{10}{\mathrm{urine}}_{\mathrm{time}},$$

where: ${aMT6sratio}_{ID}$ is the natural log of aMT6s/creatinine per visit; X_1_ is the natural log CME mass, energy or speed.

Visits where persons reported melatonin use the night before urine collection were excluded in addition to when the aMT6sr was above the upper 95th percentile (aMT6sr > 77.8), suggesting unreported melatonin use (from 143 patients, 3 were excluded). That is consistent with the upper limit of physiologic levels reported in the literature [12]. We assessed the effect modification by obesity (BMI > 30), diabetes, and %-predicted pre-bronchodilator FEV_1_ < 50% and ≥ 50% predicted using multiplicative interaction terms and stratified effect estimates. Effects on aMT6sr were calculated by multiplying each beta and corresponding 95% confidence interval values by log IQR of CME mass, speed and energy and exponentiating. After subtracting 1 from each estimate and multiplying by 100, the results are expressed as percent increase in aMT6sr per log IQR of each exposure. We assessed the correlation among the exposure variables (Additional file 1) by calculating Pearson and Spearman correlation coefficients (Additional file 1). We also examined each model residuals to assess model fit. We performed all analyses using SAS 9.4 software.

## Results

### Characteristics of the study population

After excluding visits with aMT6sr above the upper 95th percentile, there were 140 participants with 440 visits (75 patients with 4 visits, 25 patients with 3 visits, 25 patients with two visits, and 15 patients with only one visit) and approximately 95% of the visits were completed over one year. The clinical characteristics of the patients are showed in the Table 1. Most of them were elderly white men, having a mean age of 72.7 (SD = 8.2) years. Thirty-six percent (25.7%) reported diabetes, and 66(47.2%) were obese at study entry. There were 73 (52.1%) patients using beta blockers, and 21(15%) using non-steroidal taking anti-inflammatory medication (Table 1). aMT6sr levels varied widely among study participants [mean = 14.8 (SD = 11.4); median = 11.2 (IQR = 11.7)]. There was a strong correlation among CME parameters (R > 0; *p*-value > 0.05) (details in the Additional file 1).

**Table 1:** Characteristics of patients with COPD between 2013 to 2017

	First visit	Overall
	n = 140	n_obs_ = 440
Age (years)	72.7 (8.2)	73.1 (8.3)
BMI (kg/m^2^)	30 (5.7)	30.3 (6.1)
Past lifetime smoking (packyears)	59.9 (5.7)	58.3 (37.1)
Pre-bronchodilator % predicted FEV_1_	64.5 (21.9)*	64.7 (22)**
Pre-bronchodilator % predicted FVC	85.5 (20.2)*	85 (20.5)**
FEV_1_/FVC ratio	0.54 (0.13)*	0.55 (0.12)**
aMT6s (ng/ml)	15.6 (13.9)	17.4 (18.2)
Log-aMT6sr	2.5 (0.7)	2.5 (0.7)
Creatinine (mg/dl)	112.1 (57.4)	120 (68.9)

	N (%)	N (%)
Race		
White	125 (89.3)	390 (88.6)
Non-white	15 (10.7)	50 (11.4)
Sex		
Female	4 (2.8)	11 (2.5)
Male	136 (97.2)	429 (97.5)
Season		
Winter	21 (15)	101 (23)
Spring	35 (25)	103 (23.4)
Summer	48 (34.3)	121 (27.5)
Fall	36 (25.7)	115 (26.2)

Comorbidities	N (%)	N (%)
Reduced lung function (pre-bronchodilator FEV_1_ < 50% predicted)***	35 (25.8)	112 (25.9)
Obesity (BMI > 30)	66 (47.2)	209 (47.5)
Diabetes	36 (25.7)	113 (25.7)
Sleep apnea	42 (30)	139 (31.6)

Medications (within 1 day of urine collection)	N (%)	N (%)
Beta blocker	73 (52.1)	232 (52.7)
Non-steroidal anti-inflammatory medication	21 (15)	61 (13.8)

Solar activity assessment [Coronal Mass Ejection (CME)]	Mean (SD)	Mean (SD)
Log-CME_speed_	7.3 (0.4)	7.2 (0.5)
Log-CME_mass_	35.8 (0.8)	35.6 (0.9)
Log-CME_energy_	70.2 (1.2)	69.9 (1.3)
CME_speed_	1519.4 (627.2)	1503.6 (694.9)
CME_mass_	4.2 × 10^16^ (2.5 × 10^16^)	3.8 × 10^15^ (2.6 × 10^15^)
CME_energy_	5.1 × 10^30^ (5.3 × 10^30^)	4.5 × 10^30^ (5.4 × 10^30^)

*N = 136, **N = 433, ***based on Hankinson et al. [24]

### Primary analysis

**Fig. 1:**
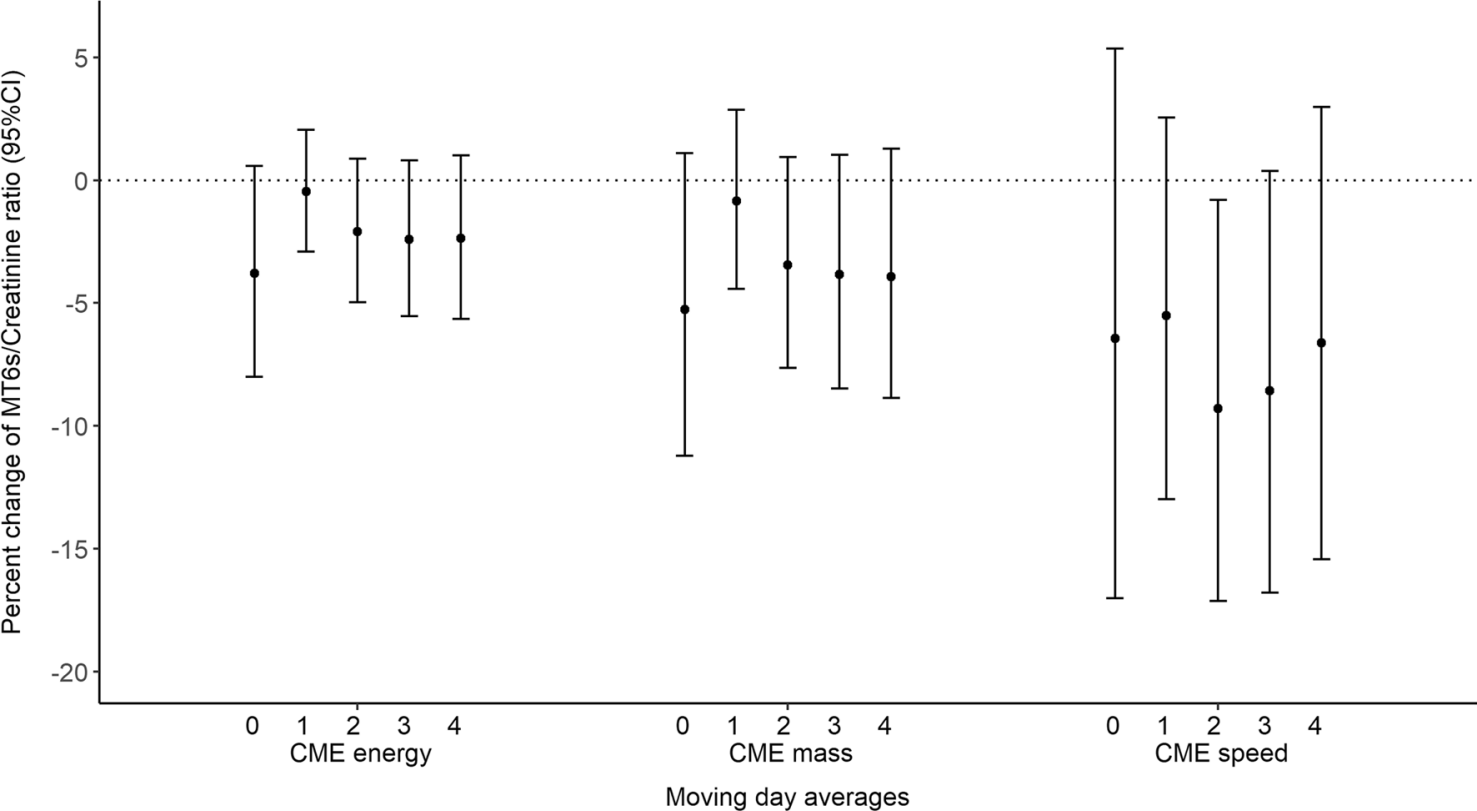
Associations of natural log CME (energy, mass, and speed) with aMT6s/Creatinine ratio. The primary models included a single exposure variable (log corona mass ejection energy, speed or mass) and model covariates

There was inverse relationship between each CME parameter moving average (from day 0 to 4 days prior to the urine collection) and creatinine-adjusted melatonin, consistent with an association between intense solar particle events and reduction in aMT6sr (Fig. 1). The point estimates for CME_speed_ were more negative than those of the other CME parameters, indicating a slightly greater reduction (Fig. 1). For an increase of an IQR of 1.1 (2.1 km/s) in natural log CME_speed_ two days prior to the day of examination, there was a reduction of 9.3% (95%CI: − 17.1%, − 0.8%; *p*-value: 0.02) or − 0.1 units (95%CI: − 0.2; − 0.01) of natural log-transformed aMT6sr levels (Fig. 1; Additional file 1).

### Effect modification

**Fig. 2:**
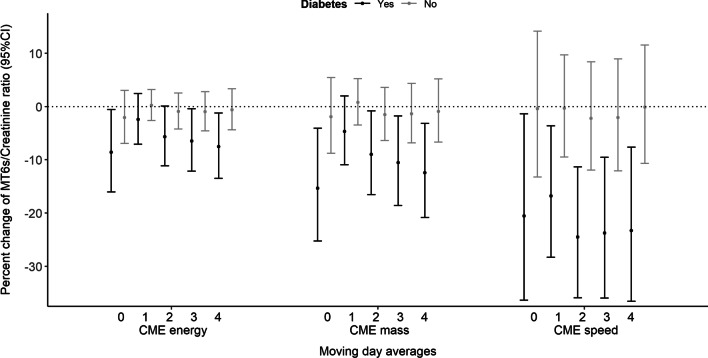
Associations of natural log CME with aMT6s/creatinine ratio modified by diabetes. Effect modification of diabetes. The models included a single exposure variable (log corona mass ejection energy, speed or mass) and model covariates

There were greater CME-related effects in patients with diabetes mellitus (*p*-interaction = 0.006) (Fig. 2; Additional file 1). For example, an IQR of 1.1 (2.1 km/s) natural log CME_speed_ two days prior to the sample collection was associated with a reduction of 24.5% (95%CI: − 35.9%, − 11.6%; *p*-value: 0.0006) or − 0.3 units (95%CI: − 0.5, − 0.1) in natural log aMT6sr levels in patients with diabetes. In patients without diabetes there was no association [aMT6sr: − 2.2% (95%CI: − 12%, 8.4%; *p*-value: 0.66) or − 0.02 natural log units (95%CI: − 0.1, 0.1] (Fig. 2; Additional file 1). There was no evidence of effect modification attributable to obesity and reduced pulmonary function (Additional file 1).

## Discussion

To the best of our knowledge, this is the first study to demonstrate a reduction of aMT6sr during periods of intense high-energy solar particle events. The associations were stronger and more robust in patients with diabetes mellitus. Although all CME parameters are strongly correlated and represent the ejection of high energy solar material, the association between CME speed and MT6sr was greater than other parameters. As CME_speed_ parameter seems to play a critical role on the atmospheric photoionization processes and energetic particle precipitation, and on Earth’s magnetic field disturbances, these factors may be linked for the observed impact on melatonin levels [23]. Intense solar activity can impact human health directly, by the modulation and disruption of the 24 h-circadian rhythm, and indirectly by inducing physicochemical properties and toxicity of atmospheric aerosols [9, 12].

Solar activity continuously emits a broad-spectrum range of electromagnetic radiation that modulates changes in the solar–terrestrial environment over time scales ranging from minutes to millennia. Sun-Earth interaction plays major roles in the radioactive, physiochemical processes and dynamics of Earth systems, which affect the human health possibly by the reduction of melatonin synthesis and ANS dysregulation related to the disruption of the 24 h circadian rhythm located in the SCN [15]. While it is unclear why patients with diabetes were more susceptible to the detrimental impact of intense solar activity on aMT6sr levels, the literature describes a link between aMT6s and diabetes risk [4, 7, 17]. Reduced levels of melatonin can impair diabetes management and disrupt blood sugar control [7], which suggests that diabetic patients may experience periods of poor health during intense solar activity periods.

A bidirectional relationship between melatonin levels and insulin secretion may explain our findings in patients with diabetes. The disruption of the 24 h circadian rhythm with subsequent lower nocturnal melatonin secretion is associated with insulin secretion and resistance [4, 17], which may influence the development and progression of type 2 diabetes [4]. The circadian rhythm regulates the sleep/wake and feeding/fasting cycles through melatonin, impacting glucose homeostasis by influencing the timing of insulin secretion from pancreatic β cells, glucose production by the liver, insulin-dependent glucose GLUT 4 expression in skeletal muscles, among other effects [4]. There is a synchronization between insulin levels and melatonin during the night and day, in which melatonin action is inhibited by insulin release (and reduce glucose tolerance) through its membrane receptors MT_1_ and MT_2_, and the secondary messengers 3′,5′-cyclic adenosine monophosphate, 3’,5’-cyclic guanosine monophosphate, and inositol 1,4,5-trisphosphate [6]. High levels of insulin are observed when melatonin levels are low, and during the night low levels of insulin are observed with high levels of melatonin [6]. Reduction of the melatonin receptor MT_2_ may play a role on the development of type 2 diabetes and metabolic diseases [6]. Concomitantly, age-related reduction levels of melatonin accompany an increase of age-related insulin resistance and type 2 diabetes risk [6]. Patients with both diabetes and COPD appear to be more susceptible to solar activity reducing circulating melatonin, and potentially antioxidant effects of melatonin. This finding suggests that differences in the response to solar activity could in part explain a greater susceptibility to effects of illnesses and environmental exposures that promote oxidative stress in patients with diabetes [25].

This study has limitations and strengths. As the original COPD study was not designed to assess effects linked to melatonin, the time of urine collection was not standardized and there is no information about melatonin determinants such as sleep habits and nocturnal light exposures, and the cohort included mainly white males. However, we improved our estimation by adjusting our models for covariates linked to impaired melatonin secretion, including urine collection time, sleep apnea history and treatment, beta blocker use, and diabetes history as covariates. As melatonin excretion is greatest at night, urine from a first morning void might be more informative, while 24-h urine collection would be ideal to assess overall impact on melatonin secretion. This limitation should bias towards a null result; therefore, the effect that we observed is likely underestimated. As melatonin was assessed at only one time of day, we are unable to comment on the effects of solar activity on circadian rhythm per se, which would require repeated measures across the day. Moreover, our findings may not be generalized to other populations. Our study strengths are the availability of this cohort with stored urine samples for every visit, extensive information regarding personal and clinical characteristics, which created a unique opportunity to test our hypotheses.

## Conclusions

Our findings contribute to understanding relationships between solar activity and susceptibility to disruption to 24 h-circadian rhythm that result in lower levels of melatonin, a circulating anti-oxidant. These results provide evidence for a biologic pathway related to intense solar activity that may be responsible for adverse health effects. Hence, our study findings have critical relevance to understand the impact of the periodicity of solar activity intensity on aMT6sr levels in high risk patients with COPD, which impacts the progression and prognosis of the disease and comorbidities.

## Supplementary Information

**Additional file 1: Table S1** Supplementary descriptive analysis. **Table S2** Pearson correlation analysis [is this for n=440? Show Pearson for natural log as this is what you use in the models. **Table S3** Associations of log CME with aMT6s/Creatinine modified by pre bronchodilator %-predicted FEV_1_, obesity, and diabetes in patients with COPD. Results expressed as % -change per overall IQR of log CMEspeed, CMEenergy, and CMEmass for each moving average starting with the day 0, the day of urine collection through 4 days before collection. **Table S4** Associations of log CME with aMT6s/Creatinine modified in patients with COPD. Results expressed as % -change per overall IQR of log CMEspeed, CMEenergy, and CMEmass for each moving average starting with the day 0, the day of urine collection through 4 days before collection. **Fig. S1**. Associations of CME_speed_, CME_energy_, CME_mass_ with MT6s/creatinine modified by obesity (BMI>30). **Fig. S2**. Associations of CME_speed_, CME_energy_, CME_mass_ with MT6s/creatinine modified by post-bronchodilator %-predicted FEV_1_ <50%.

## Data Availability

Data is available for sharing upon request in the setting of an approved IRB protocol and executed data sharing agreement.

## Abbreviations

COPD: Chronic obstructive pulmonary diseases
aMT6s: Urinary 6-sulfatoxymelatonin
aMT6sr: Creatinine-adjusted aMT6s
CME: Coronal mass ejection
CME_energy_: Energy of CME in in *erg*,* unit* of energy equal to 10^−7^ J
CME_mass_: Mass of CME in grams of solar mass
CME_speed_: Speed of CME in km/s
SCN: Suprachiasmatic Nucleus
SGA: Solar and geomagnetic activity
TNF-α: Tumor necrosis factor alpha
VA: Veterans Affairs
FEV1: Bronchodilator forced expiratory volume in one second
FVC: Forced vital capacity
FEV_1_/FVC: FEV_1_/FVC ratio
IQR: Interquartile range
BMI: Body Mass Index
ANS: Autonomic nervous system
MT: Melatonin receptors
