# Supplementary material for: Intense solar activity reduces urinary 6-sulfatoxymelatonin in patients with COPD
Source: Respir Res. 2023 Mar 24;24:91. doi: 10.1186/s12931-023-02390-w (PMC10037776; doi:10.1186/s12931-023-02390-w)
Supplement: Supplementary file 1 — Additional file 1: Table S1 Supplementary descriptive analysis. Table S2 Pearson correlation analysis [is this for n=440? Show Pearson for natural log as this is what you use in the models. Table S3 Associations of log CME with aMT6s/Creatinine modified by pre bronchodilator %-predicted FEV1, obesity, and diabetes in patients with COPD. Results expressed as % -change per overall IQR of log CMEspeed, CMEenergy, and CMEmass for each moving average starting with the day 0, the day of urine collection through 4 days before collection. Table S4 Associations of log CME with aMT6s/Creatinine modified in patients with COPD. Results expressed as % -change per overall IQR of log CMEspeed, CMEenergy, and CMEmass for each moving average starting with the day 0, the day of urine collection through 4 days before collection. Fig. S1. Associations of CMEspeed, CMEenergy, CMEmass with MT6s/creatinine modified by obesity (BMI>30). Fig. S2. Associations of CMEspeed, CMEenergy, CMEmass with MT6s/creatinine modified by post-bronchodilator %-predicted FEV1 <50%. [file 12931_2023_2390_MOESM1_ESM.docx]

**Intense solar activity reduces urinary 6-sulfatoxymelatonin levels secretion in patients with Chronic Obstructive Pulmonary Disease**

**Supplementary tables**

1ST. Supplementary descriptive analysis

| **Variable** | **N** | **Mean** | **Std Dev** | **Minimum** | **Maximum** |
| --- | --- | --- | --- | --- | --- |
| **log CMEspeed** | 440 | 7.208063 | 0.477366 | 5.666463 | 8.523223 |
| **logCMEenergy** | 440 | 69.94344 | 1.273668 | 64.59697 | 72.74864 |
| **logCMEmass** | 440 | 35.61598 | 0.842056 | 30.83372 | 37.35833 |

log = natural log

aMT6s = 6-sulfatoxymelatonin

CME = Corona Mass Ejection

| **Simple Statistics** | | | | | | |
| --- | --- | --- | --- | --- | --- | --- |
| **Variable** | **N** | **Mean** | **Std Dev** | **Median** | **Minimum** | **Maximum** |
| **Pack years** | 440 | 58.27 | 37.04 | 49.88 | 10 | 212 |
| **Age** | 440 | 73.03 | 8.31 | 72.69 | 46.68 | 90 |
| **BMI** | 440 | 30.25 | 6.1 | 29.73 | 15.56 | 50.84 |
| **aMT6s** | 440 | 17.39 | 18.2 | 11.69 | 0.11 | 167.25 |
| **aMT6sr** | 440 | 14.86 | 11.43 | 11.18 | 0.6 | 77.76 |
| **% predicted fev1** | 433 | 64.71 | 21.95 | 65.56 | 15.69 | 122.49 |
| **% predicted fvc** | 433 | 84.97 | 20.51 | 83.07 | 35.71 | 144.78 |
| **ratio** | 433 | 0.55 | 0.13 | 0.56 | 0.22 | 0.91 |

2ST. Pearson correlation analysis

|  |  | **CMEspeed** | **CMEenergy** | **CMEmass** |
| --- | --- | --- | --- | --- |
| **CMEspeed** | **R** | 1 | 0.84979 | 0.79973 |
|  | *p*-value |  | <.0001 | <.0001 |
| **CMEenergy** | **R** | 0.84979 | 1 | 0.95441 |
|  | *p*-value | <.0001 |  | <.0001 |
| **CMEmass** | **R** | 0.79973 | 0.95441 | 1 |
|  | *p*-value | <.0001 | <.0001 |  |

3ST. Associations of log CME with aMT6s/Creatinine modified by pre bronchodilator %-predicted FEV_1_, obesity, and diabetes in patients with COPD. Results expressed as % -change per overall IQR of log CMEspeed, CMEenergy, and CMEmass for each moving average starting with the day 0, the day of urine collection through 4 days before collection.

Footnote Fig.3ST. Effect modification of comorbidities (diabetes, obesity,pre-bronchodilator percent predicted FEV_1_ <50%). The models included a single exposure variable (log corona mass ejection energy, speed or mass) and covariates. Model covariates included *a priori* were race (white vs. other), sex (male/female), age, body mass index (BMI), beta blockers taken within 1 day of urine collection, non-steroidal anti-inflammatory medication taken within 1 day of urine collection diabetes, time of urine collection, history of sleep apnea, and season (winter, spring, summer, fall). Significant results: In bold letters (*) *p*-value<0.05

4ST. Associations of log CME with aMT6s/Creatinine modified in patients with COPD. Results expressed as % -change per overall IQR of log CMEspeed, CMEenergy, and CMEmass for each moving average starting with the day 0, the day of urine collection through 4 days before collection.

|  |  |  | β | IQR | % | Min% | Max% | p-value |
| --- | --- | --- | --- | --- | --- | --- | --- | --- |
| COPD cohort | CMEspeed | 0 | -0.061 | 1.088 | -6.438 | -17.018 | 5.357 | 0.272 |
|  |  | 0-1 | -0.050 | 1.120 | -5.502 | -12.982 | 2.549 | 0.175 |
|  |  | 0-2 | -0.087 | 1.110 | **-9.290** | **-17.128** | **-0.799** | **0.033** |
|  |  | 0-3 | -0.081 | 1.100 | -8.572 | -16.797 | 0.381 | 0.060 |
|  |  | 0-4 | -0.062 | 1.110 | -6.628 | -15.433 | 2.989 | 0.170 |
|  | CMEenergy | 0 | -0.035 | 1.100 | -3.794 | -8.003 | 0.589 | 0.089 |
|  |  | 0-1 | -0.004 | 1.020 | -0.456 | -2.900 | 2.048 | 0.718 |
|  |  | 0-2 | -0.020 | 1.030 | -2.082 | -4.964 | 0.885 | 0.167 |
|  |  | 0-3 | -0.024 | 1.030 | -2.414 | -5.535 | 0.807 | 0.140 |
|  |  | 0-4 | -0.023 | 1.030 | -2.369 | -5.647 | 1.018 | 0.168 |
|  | CMEmass | 0 | -0.053 | 1.025 | -5.252 | -11.217 | 1.103 | 0.103 |
|  |  | 0-1 | -0.008 | 1.028 | -0.838 | -4.415 | 2.868 | 0.652 |
|  |  | 0-2 | -0.033 | 1.048 | -3.441 | -7.638 | 0.935 | 0.121 |
|  |  | 0-3 | -0.038 | 1.041 | -3.833 | -8.473 | 1.031 | 0.120 |
|  |  | 0-4 | -0.038 | 1.039 | -3.917 | -8.868 | 1.291 | 0.137 |

Footnote Fig.4ST. The models included a single exposure variable (log corona mass ejection energy, speed or mass) and covariates. Model covariates included *a priori* were race (white vs. other), sex (male/female), age, body mass index (BMI), beta blockers taken within 1 day of urine collection, non-steroidal anti-inflammatory medication taken within 1 day of urine collection diabetes, time of urine collection, history of sleep apnea, and season (winter, spring, summer, fall). Significant results: In bold letters (*) *p*-value<0.05

**Supplementary figures**

Fig. 1s. Associations of CME_speed_, CME_energy_, CME_mass_ with MT6s/creatinine modified by obesity (BMI>30).


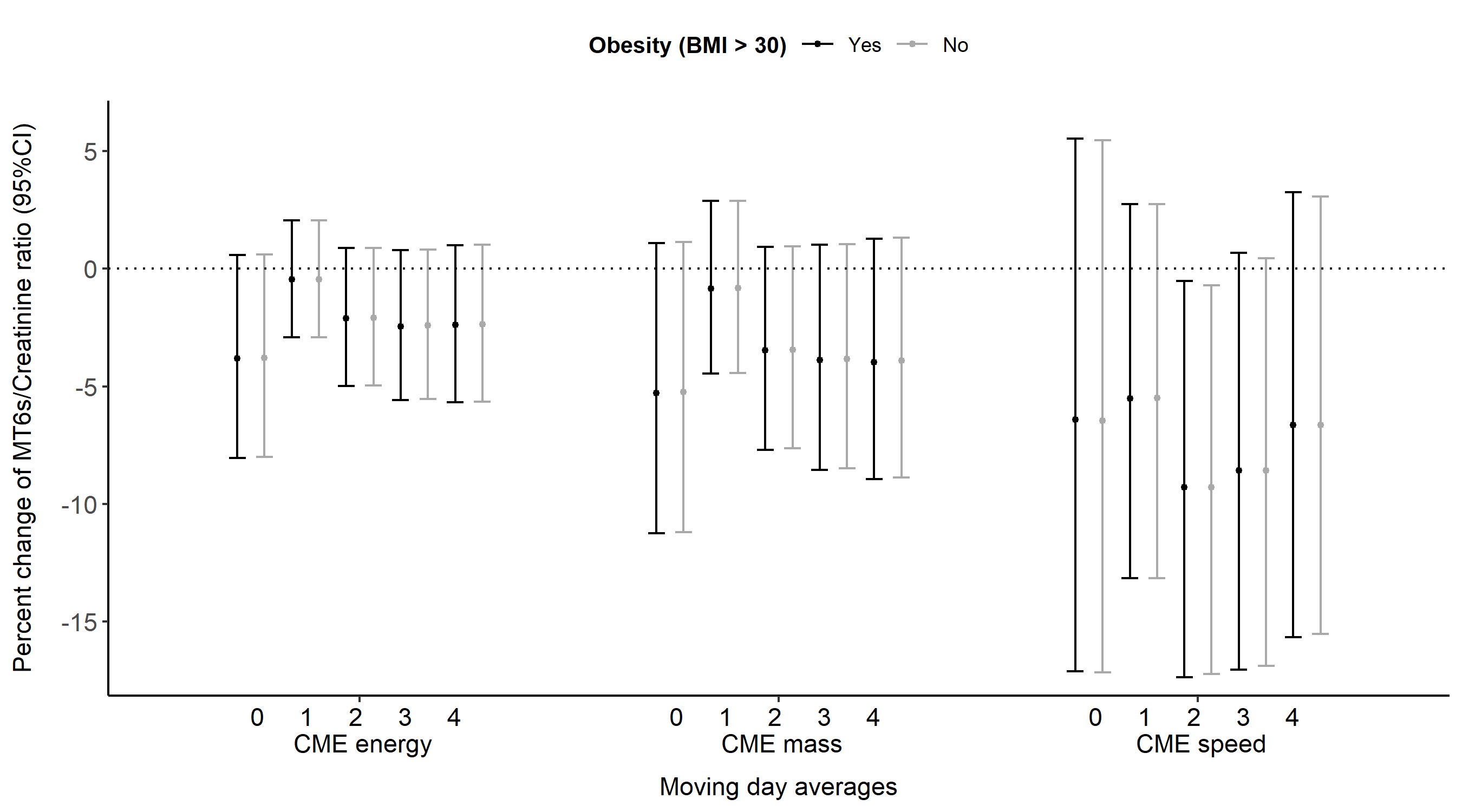


Footnote Fig.1s. The models included a single exposure variable (logged corona mass ejection energy, speed or mass) and covariates.

Fig. 2s. Associations of CME_speed_, CME_energy_, CME_mass_ with MT6s/creatinine modified by post-bronchodilator %-predicted FEV_1_ <50%.


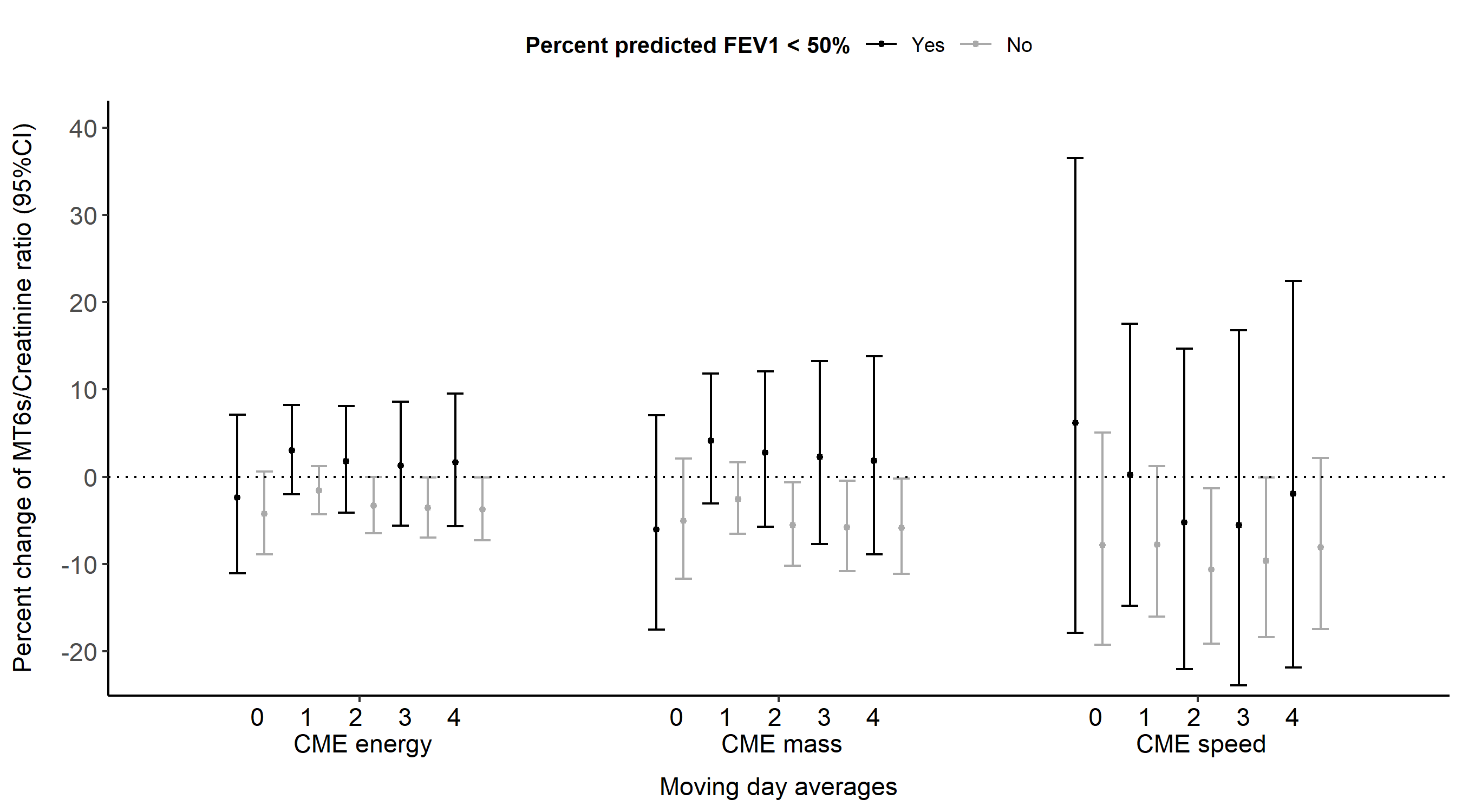


Footnote Fig.2s. Black and grey colors: Effect modification of percent predicted FEV_1_ <50%. The models included a single exposure variable (logged corona mass ejection energy, speed or mass) and covariates.
